# Supplementary material for: SPOP expression is associated with tumor-infiltrating lymphocytes in pancreatic cancer
Source: PLoS One. 2024 Jul 29;19(7):e0306994. doi: 10.1371/journal.pone.0306994 (PMC11285963; doi:10.1371/journal.pone.0306994)
Supplement: S1 Fig — (A-I) the ROC curve of Bladder Urothelial Carcinoma (BLCA), Cervical squamous cell carcinoma and endocervical adenocarcinoma (CESC), Acute Myeloid Leukemia-like (LAML), Lung squamous cell carcinoma (LUSC), Ovarian serous cystadenocarcinoma (OV), Pancreatic adenocarcinoma (PAAD), Thymoma (THYM), Uterine Corpus Endometrial Carcinoma (UCEC) and Uterine Carcinosarcoma (UCS). (DOCX) [file pone.0306994.s001.docx]

Title: *SPOP Expression Is Associated with tumor-infiltrating lymphocytes in Pancreatic Cancer*

Xiao juan Yang^1^, Yong Feng Xu^1^, Qing Zhu^1^*


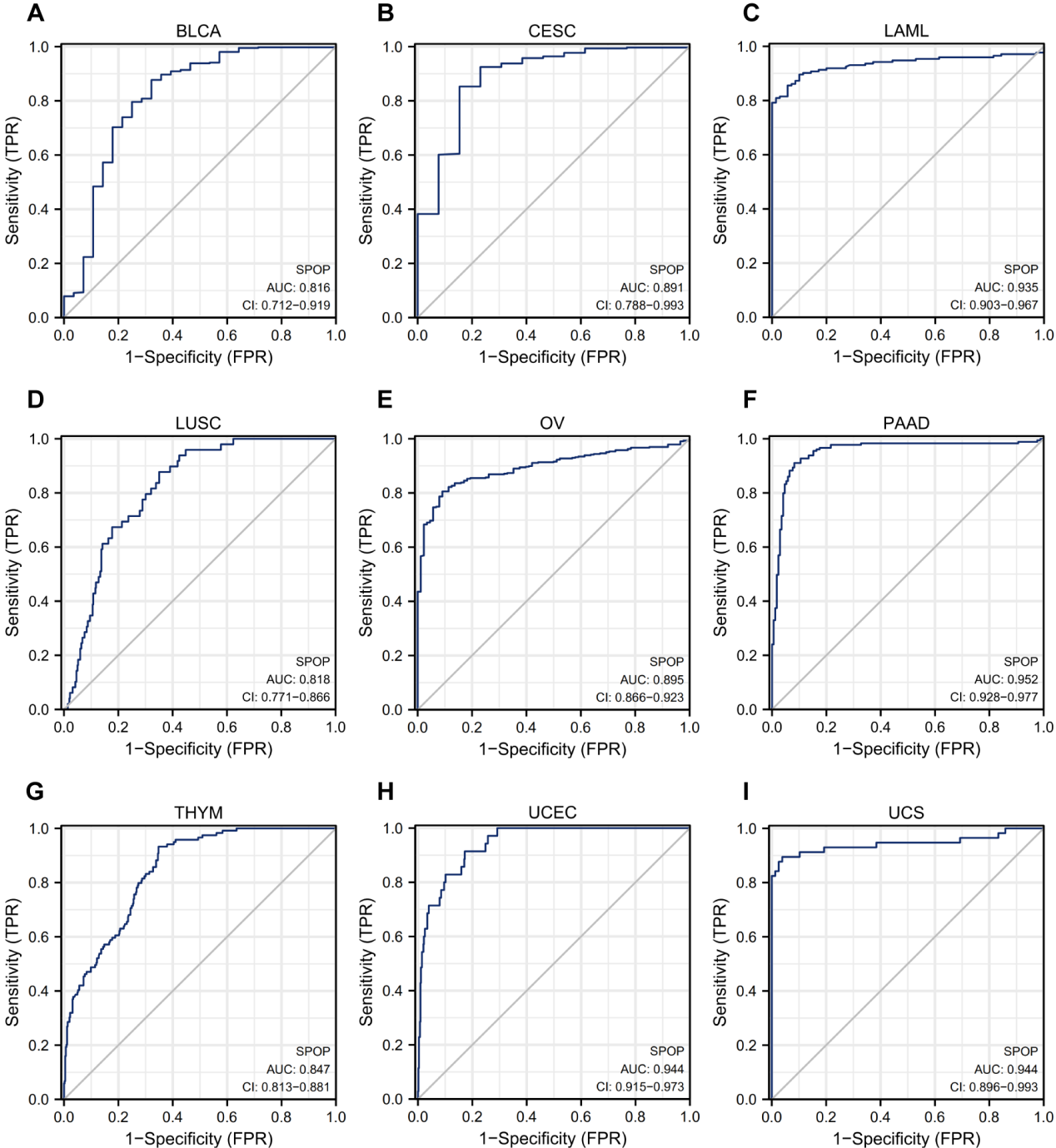


**Figure S1. The AUC were performed in types of cancer.** (A-I) the ROC curve of Bladder Urothelial Carcinoma (BLCA), Cervical squamous cell carcinoma and endocervical adenocarcinoma (CESC), Acute Myeloid Leukemia-like (LAML), Lung squamous cell carcinoma (LUSC), Ovarian serous cystadenocarcinoma (OV), Pancreatic adenocarcinoma (PAAD), Thymoma (THYM), Uterine Corpus Endometrial Carcinoma (UCEC) and Uterine Carcinosarcoma (UCS).
